# A Traditional Scientific Perspective on the Integrated Information Theory of Consciousness

**DOI:** 10.3390/e23060650

**Published:** 2021-05-22

**Authors:** Jon Mallatt

**Affiliations:** The University of Washington WWAMI Medical Education Program at The University of Idaho, Moscow, ID 83844, USA; jmallatt@uidaho.edu

**Keywords:** theories of consciousness, recurrent interactions, causal structure, mind-body problem, evidence, neurobiological naturalism, neuroevolution, scientific theory/method, inductive versus deductive inference, artificial intelligence, neural correlates of consciousness

## Abstract

This paper assesses two different theories for explaining consciousness, a phenomenon that is widely considered amenable to scientific investigation despite its puzzling subjective aspects. I focus on Integrated Information Theory (IIT), which says that consciousness is integrated information (as **ϕ**^Max^) and says even simple systems with interacting parts possess some consciousness. First, I evaluate IIT on its own merits. Second, I compare it to a more traditionally derived theory called Neurobiological Naturalism (NN), which says consciousness is an evolved, emergent feature of complex brains. Comparing these theories is informative because it reveals strengths and weaknesses of each, thereby suggesting better ways to study consciousness in the future. IIT’s strengths are the reasonable axioms at its core; its strong logic and mathematical formalism; its creative “experience-first” approach to studying consciousness; the way it avoids the mind-body (“hard”) problem; its consistency with evolutionary theory; and its many scientifically testable predictions. The potential weakness of IIT is that it contains stretches of logic-based reasoning that were not checked against hard evidence when the theory was being constructed, whereas scientific arguments require such supporting evidence to keep the reasoning on course. This is less of a concern for the other theory, NN, because it incorporated evidence much earlier in its construction process. NN is a less mature theory than IIT, less formalized and quantitative, and less well tested. However, it has identified its own neural correlates of consciousness (NCC) and offers a roadmap through which these NNCs may answer the questions of consciousness using the hypothesize-test-hypothesize-test steps of the scientific method.

## 1. Introduction

Integrated Information Theory (IIT) is a major theoretical framework for investigating the phenomenal experiences of consciousness. It “attempts to identify the essential properies of consciousness (*axioms*) and from there, infers the properties of physical systems that can account for it (*postulates*)” [1]. It explains both the quality and quantity of consciousness that is intrinsic to a system, as derived from first principles [1,2,3]. It says consciousness is integrated information, specifically the maximum amount of integrated information a system has. Integrated information (quantified as **ϕ**) is the information specified by a whole system over and above that which is specified by its component parts. Therefore, integrated information is a measure of the back-and-forth (recurrent) *interactions* between the parts. Causation is a key component of IIT. In particular, IIT quantifies the cause-effect power of the system and its parts upon themselves. If the causes and effects of a system or a mechanism are reducible to those of its parts alone—meaning the parts do not interact—then its integrated information is zero. IIT is unique among the major theories of consciousness in starting with the phenomenal and proceeding to the physical. Once more, it starts with axioms about experience and then makes postulates that explain how the physical elements relate to an experience (see Note 1 in Appendix A).

I will assess IIT from my perspective as an evolutionary neuroscientist, covering its strong and weak points as I see them. In so doing, I introduce some angles that have not been considered in past appraisals of the theory. After evaluating IIT, I will compare it to another theory of consciousness, named Neurobiological Naturalism (NN), which was developed by Todd E. Feinberg and myself [4,5,6]. NN was devised to explain how consciousness evolved and how its subjective aspects could have emerged in our physical world. It seeks these answers by identifying the neural features that always associate with consciousness, called neural correlates of consciousness or NCCs [7], [8] (pp. 81–95), [9], so that scientists can work out how and which NCCs “cause” consciousness. Proceeding from the physical to the phenomenal via the NCCs, NN follows a common approach in consciousness studies and is, as we will see, different from the approach taken by IIT.

Here are some initial comparisons. NN matches IIT by emphasizing the importance of recurrent interactions between parts—feedback responses to feedforward signals—as do many other theories of consciousness [10,11,12,13,14]. Both theories recognize the standard definitions of consciousness, such as the awareness of one’s external and internal existence [15], and both focus on the most basic form of consciousness, called primary consciousness, which is raw experience without reflection [3,16,17]. Like other researchers who work on IIT [18], I synonymize the terms, “consciousness”, “experiences”, “feelings”, “subjective experiences”, and “sentience” [19]. However, IIT *additionally* defines consciousness as the integrated information in a physical system, and that makes it different from other theories. It says more integrated information means more consciousness. More on this below.

IIT and NN have similar goals in that they both seek to understand the puzzling subjective aspects of consciousness, as well as the *physical* substrates that are responsible for those aspects. Both theories are physicalist in that respect.

IIT and NN were constructed in different ways. IIT is a deductive theory because it goes from general axioms to the theory itself, to its specific testable predictions. NN is an inductive theory that goes from specific observations to generalizations, to the theory and its tests. I will consider whether these different constructions make a difference in the relative strengths and weaknesses of the theories, in a way that can improve future investigations in the field of consciousness studies.

## 2. Integrated Information Theory

### 2.1. Strengths of IIT

IIT’s first strength is that its five axioms seem to characterize human experience well. These axioms are intrinsic existence, composite nature, information, integration, and exclusion [3,9,20]. *Intrinsic existence* means that only the subject has this experience, and no outsider observer can. *Composite nature* means an experience is a structured assembly of distinct components, such as green, grass, stadium, soccer ball, cheers. *Specific information* means the experience has a defined information content that distinguishes it from every other experience that the subject could have. *Integration* means consciousness is integrated and not divisible into distinct subsets of experience: one sees the entire, integrated visual field and cannot subdivide this into separate experiences of the field’s left and right halves. *Exclusion* means every experience has a definite content, no more or less, and flows into the next experience at a definite speed, no slower or faster. These axioms cover all the phenomenal properties I can think of (for a different opinion, see [21]).

A second strength is IIT’s strong logic, rigor and mathematical formalism, especially since it was upgraded to Version 3.0 in 2014 [2]. Third, IIT is clever to use the reversed perspective of starting with the phenomenal rather than the physical. I find this to be a creative and valuable way to study consciousness. One benefit of phenomenology-first is that it avoids the puzzling “hard problem” [22] or explanatory gap [23] that comes from the traditional approach of starting from the physical and asking how and why physical processes can give rise to experience [1,18] (also see Note 2 in Appendix A). The reason IIT obliterates the hard problem is that it says its maximally integrated information *is identical* to consciousness. More specifically, IIT characterizes experience not as a system’s material processes or structures, but as the abstract *cause-effect power* or *causal structure* (or *conceptual structure*: see Figure 1 in [3]). IIT also solves another part of the mind-body problem: how could the mental cause bodily behavior? IIT answers this Mental Causation question by defining every system with internal causal factors as conscious [24,25].

A fourth strength of IIT is that it does a good job of accommodating organic evolution. Both Darwinian natural selection and IIT yield systems with high operational efficiency. This is seen in IIT’s tenet that consciousness is limited to the part of the system with a *maximum* of integrated information, called a maximally irreducible cause-effect structure (MICS) or **ϕ**^Max^ (see Note 3 in Appendix A). IIT says that consciousness increased during brain evolution because it provides an adaptive advantage, specifically that “integrated neural networks are more efficient [than feedforward-only architectures] in terms of the amount of functions that can be specified by a given number of neurons and connections;” and adaptive evolution assures “that the intrinsic cause-effect structure of an organism [i.e., of its brain] “matches” the causal structure of its environment,” thus favoring survival [26] (p. 98). As an example of this match, statistical regularities in one’s complex environment, such as “lethal predators come to my water hole every day at dusk”, can become incorporated into the brain’s cause-effect structure by natural selection [3] (p. 458), [9] (p. 122). In addition, computer simulations called “animats” were found to evolve more internal connectivity (more integrated information) over the generations when they were subjected to more difficult and complex environments [25,27,28].

The value of linking IIT to evolution is revealed by a recent study that *missed* this link. Namely, Doerig et al. [29] tried to falsify IIT with a behaviorist argument that, to its detriment, failed to consider the role of natural selection. This “unfolding argument” was based on the fact that purely feedforward architectures can perform any function that a recurrent network can, given the same inputs and outputs. This fact also holds for any hybrid system with both feedforward and recurrent connections. Given this, the unfolding argument reasons as follows: when one function can result from so many different neural mechanisms or “causal structures,” no empirical evidence could ever tell us what the actual, specific causal structure was; i.e., the particular structure that produced the measured output in response to a particular input. Thus IIT, which depends on identifying a single causal structure, was said to fail. However, the unfolding argument missed the key fact that purely feedforward architectures are far less efficient and less compact than recurrent systems, and also are more energy-expensive and prone to breakdown [9] (pp. 142–143). They are so much less effective that natural selection would never let such feedforward systems evolve. Perhaps the unfolding argument could apply in some idealized world that is based only on logic, but not in the dangerous and competitive world of reality. Tsuchiya et al. [30] pointed out other difficulties with the unfolding argument but not this evolutionary one, which seems the strongest rebuttal.

A fifth strength of IIT is that its proponents have deftly rebutted claims that it is panpsychism [31,32], [33] (p. 11)—panpsychism being the theory that consciousness is ubiquitous in the material world. IIT’s rebuttal is that objects without interacting parts are not conscious (e.g., an immobile bag of marbles), nor are those parts of any conscious system whose integrated information is less than **ϕ**^Max^ [26] (p. 99), [34] (p. 11). This rebuttal also points out that panpsychism is vague or barren because it treats consciousness as a uniform force and therefore does not differentiate between the consciousnesses of different systems, of atoms versus brains. By contrast, IIT makes these distinctions and it can give consciousness the rich structure of intrinsic existence, composite nature, information, integration, and exclusion [9] (p. 162). These arguments rebut the claim that IIT is a traditional, strict panpsychism. Other versions of panpsychism are looser in that they say *only some* basic entities have consciousness [31,35], and IIT would fit those versions. This is discussed below in Section 2.2.2.

A sixth strength is that IIT has made some impressive predictions, supported by evidence. IIT also provides reasonable explanations for otherwise puzzling observations. Some of these predictions and explanations are not specific to IIT but instead are shared by all the theories that emphasize recurrent neuronal actions: for example, the explanation for why split human brains have dual consciousnesses, and why the cerebral cortex is conscious but the cerebellum is not [36]. But other predictions of IIT are most impressive because they fit only IIT, or at least were never thought up until IIT put them forth: the features of slow-wave sleep, of anesthetization, epileptic seizures [3] (p. 458), [9] (pp. 101–104); and using the Perturbational Complexity Index to predict whether unresponsive brain-injured patients are conscious and may recover [37] (but see [38]).

IIT also offers an explanation for brain injuries where the patients not only fail to perceive sensed objects or attributes, but also deny that these even exist (e.g., the color denial of achromatopsia [3] (p. 460)). IIT’s explanation is that a destroyed brain area has no integrated information at all, in contrast to an inactive but intact area, which registers the *absence* of certain features as a part of the overall experience so that this absence still has causal power [1] (Prediction 8). I appreciate the logic of this idea that an inactive neuron (that is intact and functional) still contributes to, for example, visually experiencing a black sky as extending in space [39] (p. 32).

A seventh strength is that IIT can inform the debate over whether *group consciousness* exists [40]. Group consciousness, which says a social group of people or a hive of bees is somehow conscious at a higher level, has always raised skepticism on the general grounds that there are too few connections or interactions among the individuals in the group [5] (p. 197), [40,41]. IIT offers a more specific reason why group consciousness is impossible [9] (pp. 163–167): the fewer interactions among the individuals bring the **ϕ** value of the whole group down below the sum of the **ϕ** values of the individual minds, so that the latter is always the MICS with **ϕ**^Max^.

### 2.2. Potential Weaknesses of IIT

#### 2.2.1. The Way IIT Was Constructed

**Figure 1:**
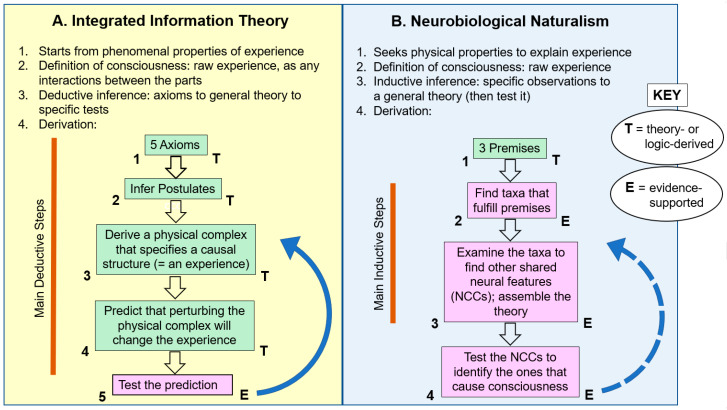
Comparing Integrated Information Theory (**A**) and the Neurobiological Naturalism Theory (**B**), especially in how they were constructed. IIT is a deductive theory built from axioms, whereas NN is an inductive theory built primarily from observed evidence. IIT relies more on theory (T) and uses evidence (E) late, whereas NN uses evidence earlier and throughout. In IIT most of the steps are theory- or logic-derived, but in NN most steps are evidence-supported. Thus, I argue, IIT risks errors, in being more dependent on theory than on confirming evidence. Both of the theories, however, are scientifically testable, meaning they can be evaluated empirically at all of the “E” steps. They are also *revisable*, by new evidence derived from the tests (within limits). This revisability is shown by the blue arrows. The blue arrow for Neurobiological Naturalism is dashed to show that the later steps of this theory are still to be tested. NCCs: neural correlates of consciousness.

Figure 1A summarizes how IIT was built, in flow-chart format. Its five axioms are used to infer its postulates, which are used to derive the physical complex that specifies the causal structure that is the experience (green boxes 1–3 in the figure). This leads to the predictions that perturbing the physical complex will change the experience (box 4), and the predictions are tested by such perturbations (box 5). The test generates evidence (E)—the first step in the process where empirical evidence appears—and any unexpected evidence is then used to adjust the theory for further testing (blue arrow in Figure 1A).

How does this compare with the way standard research science works? Standard science constructs its theories using lots of evidence *throughout* the building phase [42,43,44]. Science proceeds by repeatedly testing hypotheses, with each hypothesis confirmed by experimental and observational evidence. In this way, the theory is constructed step-by-step with each step upheld by data before going on to the next step. Theory construction may *start* with reasoned assumptions or premises, but these should be as few and as testable as possible. Testability is also essential for the final scientific theory that is generated, which must make falsifiable predictions that can be tested with further evidence [45]. As shown above, IIT does make testable predictions, so my questions about any weaknesses will focus on how it was constructed (Figure 1A).

The difference between IIT and standard science is that IIT is so heavily theory-based [2,26] and brings in evidence quite late. Only secondarily [46] does its complicated theoretical framework consider physical facts to test its predictions (the “E” step in Figure 1A). The theory-first approach is highly vulnerable to errors because without frequent empirical checks along the way, a single mistake, contrafactual step, or omission in the long chain of reasoning can send the theory in the wrong direction, making it wrong, period. This can especially happen for such an intricate and multifaceted phenomenon as consciousness, despite IIT’s extremely careful mode of theory construction and its mathematical rigor. (Also see Note 4 in Appendix A.).

The pitfalls of constructing a formal theory without taking the opportunity to consider empirical evidence along the way are shown by the Banach–Tarski paradox [47,48]. This is a formally derived mathematical theorum that says any solid object can be cut into a number of pieces and reassembled into two objects that are identical to the original, meaning each of the two has the *same volume* as the original. This “volume doubling”, while mathematically proven and supported by an axiom, is impossible in the real world, and one requires a knowledge of the world to resolve the paradox. It shows that even a perfectly built model can be wrong. If the derivation of IIT has such a mathematically correct but nomologically incorrect step, then the fatal error will remain forever undetected barring the offchance that it is someday uncovered by random empirical backchecking.

Specific places where its theory-heavy nature may make IIT vulnerable are that its five axioms somehow fail to include every aspect of experience or that its postulates do not incorporate these axioms fully. How can one be certain that IIT has correctly found *all* the axioms and translated “them into the necessary and sufficient conditions (postulates) for the physical substrates of consciousness” [3] (p. 460)? Tononi himself seems to have expressed uncertainty about this by saying, “even the correct set of axioms and corresponding postulates are still being developed and refined” [46]. The new axioms he considered adding—one axiom on time and another axiom on causal power—are sympathetic to IIT, but he opened the door for someone else to add other axioms that are not. Adding axioms could be lauded as the normal process for correcting and readjusting a scientific theory, but no, axioms are so foundational that a new one could topple the whole theory.

A counterargument to my methodological criticisms of IIT could point out that IIT is a deductive scientific theory—the whole point of the deductive strategy being to build the theory first then test it afterward—and deductive theories are just as valid in science as are inductive theories that begin by gathering evidence [49]. Furthermore, this counterargument goes, IIT is a mature theory that has gone through major revisions (1.0 through 3.0 [2,50,51]), more data was gathered in its early versions, and as any theory matures it naturally shifts from the data-gathering phase to the testing phase, becoming deductive. From this, the counterargument continues, the results of the tests’ experiments become the evidence that is required to confirm, refute, and adjust IIT’s theoretical steps.

I see two problems with this argument. First, although IIT initially did gather more neural evidence in its earliest incarnation of 1.0 and then did use that evidence to test the theory [50] (pp. 10–18), this young version already had long theoretical stretches that were never specifically tested with evidence: see Figures 1 and 2 in [50]. It is difficult to view these published figures without concluding that IIT has always put far more emphasis on theorizing than on supporting evidence. The two figures do not show real objects but theoretical abstractions. Second, the evidence from IIT’s test results has provided *broad*, correlative support for the theory’s main predictions, but has not specifically been used to evaluate the many theoretical steps of the model. Both these considerations show that IIT’s construction favored extensive theorizing over evidence, against the central role of evidence in research science [44]; and the fact that IIT is now in the deductive stage seems irrelevant to that.

Constructing an intricate theory without enough supporting evidence raises another problem for deductive inference. The scientific method says that the theory or hypothesis to be tested should be as simple as possible because a compound hypothesis “makes testing difficult if not impossible because while part may be true the other may not be so” [52]. Also, the shear number of component hypotheses that must be evaluated post-theory precludes any thorough testing for practical reasons.

Many other elaborate models of consciousness exist that stem more from theory than evidence. Examples are the quantum consciousness of Orch-OR theory [53], the phylogenetic refinement theory [54], the hierarchical forward model algorithm [55], adaptive resonance theory [56], the idea that the evolution of consciousness led to physiological stress responses [41] (pp. 426–439), the cemi field hypothesis [57], attention schema theory [58], and the idea that plants are conscious [59]. IIT is among the best of such theoretical-deductive models in its careful and formal construction, and as mentioned above, it makes good, testable predictions about the physical structure and function of the cerebral cortex, so it fits that part of the standard scientific procedure. But not having enough physical evidence at its *construction* stage can make a theory-driven model blind to any flaws it may have. The main reason for this is that once an elaborate theory is built from many people laboring on it over decades, there is tremendous inertia and resistance to discarding it if an error is found in its foundations. Another reason is that during the tests some evidence can always be found post hoc to support any theory, especially the most elaborate theories, which allow the most tests.

**Figure 2:**
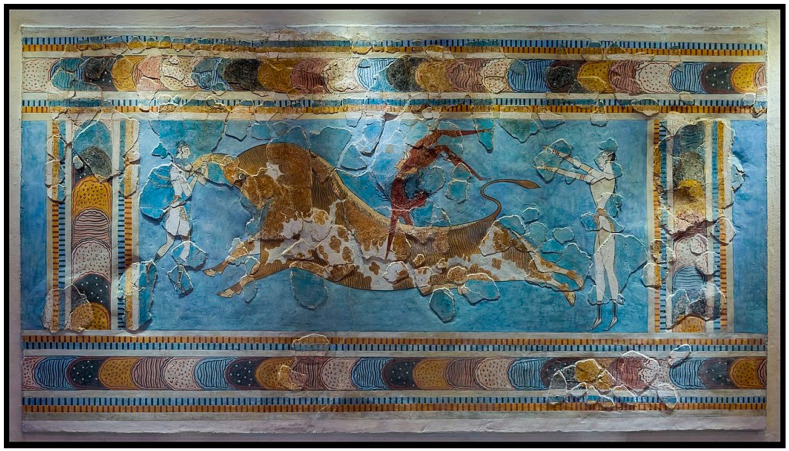
The Bull-Leaping Fresco from Minoan Crete, about 1450 BCE, as reconstructed by Émile Gilliéron. The original fragments make up less than half of it and they came from seven different wrecked panels at the archeological site of Knossos. Thus, though this reconstruction is vivid and looks coherent, one cannot tell how correct it is. Does this raise concerns about IIT, which also is based more on theory than evidence? Photo is in the public domain; see https://en.wikipedia.org/wiki/Bull-Leaping_Fresco. (accessed on 19 May 2021).

I analogize the risk faced by the elaborate, theory-over-evidence models to viewing the early twentieth century reconstructions of Bronze-Age Minoan frescos (https://edu.rsc.org/resources/restoration-of-minoan-paintings-imitation-or-reproduction/1640.article, accessed on 19 May 2021) from the fragmentary evidence that made up only a fraction of the original painting (Figure 2). The analogy is that in both cases we do not immediately realize that a lot of the physical evidence is missing, so we are likely to be misled by the reconstruction’s or model’s beauty and logic.

All these considerations on the relative importance of theorizing versus evidence in building scientific theories can reveal the best way to choose or construct a theory for explaining consciousness. In a general scientific approach there are infinitely many hypotheses/theories that can fit the observed data, and this is a problem that could slow scientific progress through inefficiency or misdirection. To solve this, scientists, as a rule, seek the best hypothesis/theory from the set by choosing the one with the simplest explanations (Occam’s razor). To this rule I add another rule for selecting the best theory, called Empirical Evaluation Delay. This rule says that theories with many model-based steps that delay testing until the theory is constructed—like IIT—are less preferred than theories that test every important step along the way, like Neurobiological Naturalism. In short, I am proposing a new method for selecting the best among candidate theories in (consciousness) science.

#### 2.2.2. Too Low a Bar for Consciousness?

IIT claims that anything with interacting parts can have consciousness (see Introduction). That includes such simple, nonliving things as one binary photodiode, a thermostat, and an isolated proton with its three interacting quarks [9] (pp. 158–163), [26,51,60]. This claim has met with a resistance that has been called “the small network argument” [33,61]. Aaronson [62] and Cerullo [36] said IIT’s claim redefines consciousness as too simple, in a way that does not match commonsense intuition or the original neuroscientific and psychological definition of “consciousness.” That traditional definition ties consciousness to a mind, a body, and sensory receptors and motor effectors that are more than just simple inputs and outputs. Tononi [46] responded to Aaronson by criticizing the use of commonsense intuition because our intuition is often wrong, so IIT is preferred. This response says that the essential properties of experience are accounted for in causal terms, so even simple things with causal effects must be conscious (Francesco Ellia, personal communication). Thus, it is a response that is consistent with IIT’s basic principles. However, “consciousness” is a core term in many fields of neuroscience so redefining it raises practical difficulties for these fields, especially because so many of the secondary terms related to consciousness would have to be redefined as well. IIT wants to integrate with the neurosciences, so might its proponents offer a solution to the terminological upset its redefinition of consciousness would cause?

A dichotomy characterizes the IIT literature. That is, IIT articles fall into two types with none in between. One class of articles treats IIT as a theory about the complex cerebral cortex (actually the cortex and the thalamus), and they either do not bring up the simple, few-component systems or else only mention them in passing (e.g., [3,30,39]). Other IIT articles, by contrast, directly cover the simpler systems as conscious [26] (p. 98), [46,51]. This dichotomy between the “simple” and “cortical” articles has a practical explanation, that **ϕ** values are only computationally tractable in simple systems [63], while the human cerebral cortex is the paradigm system for studying consciousness. However, the gap between the two study systems is large and conspicuous, so one hopes IIT will start to investigate consciousness in intermediate systems. Candidates for these intermediates are the well-characterized brain of the roundworm *Caenorhabditis elegans* and the somewhat more complex brain of larval zebrafish *Danio rerio* [64,65] (see Note 5 in Appendix A).

In contrast to many theories that say consciousness arose at some stage in the evolution of life, of animals, or of humans [66,67,68,69], IIT denies such a threshold ever existed except at the start of the universe when the first elementary particles began to interact. Since that origin, IIT maintains, the changes in consciousness have been gradual [51] (p. 236). But these changes need not have occurred at a *constant* rate. There could have been accelerations when integrated information increased markedly, during the documented evolutionary jumps in the capacities of living systems: e.g., during the evolution of the first living cells, of the first neuronal networks, first brains, and the cerebral cortex. I hope those who study IIT will consider and model these accelerations because that could reveal much about the evolution of complexity in nature.

#### 2.2.3. Affective Consciousness?

So far, the IIT studies have focused on the conscious awareness of sensory stimuli, especially vision. IIT has not, however, investigated affective feelings like emotions. Such investigations could be useful because differences exist in vertebrate brains between the regions for affective and sensory-based consciousness. For example, only the affective regions have valence neurons that code positive and negative values [70,71]. It would be interesting to know if IIT can explain how affects arise from that physical substrate. In principle, IIT could model positive and negative values within a cause-effect structure (Francesco Ellia, personal communication).

#### 2.2.4. Section Summary

A potential vulnerability in IIT is its high modeling-to-evidence ratio. A point of controversy is that it extends consciousness to relatively simple, nonliving systems.

## 3. Neurobiological Naturalism and IIT

### 3.1. Introduction and Initial Comparisons

This section compares IIT to Neurobiological Naturalism (NN), the theory of consciousness that I helped Todd Feinberg to develop [5,6,70,72,73,74,75]. NN was inspired by John Searle’s theory of Biological Naturalism [76,77], which proclaims mental events have exclusively physical causes. NN is driven by empirical evidence (see Figure 1B and Section 3.2), and it views consciousness as an emergent feature of the physical universe. NN says consciousness emerged/emerges in living *complex systems* [78,79,80,81] through a series of hierarchical levels, both over time through organic evolution and synchronically within the nervous systems of certain animals. As we will see, the theory identifies the conscious animals as all the vertebrates, all the arthropods, and cephalopod mollusks but no other organisms. The major evolutionary steps are said to have been: (1) the first living cell, as the start of *embodiment* that ultimately allowed a subjective and first-person point of view; (2) from the first nervous systems through the first brains, which laid the goundwork for brains’ role in consciousness in the next step; and (3) consciousness, from complex brains that have a set of special features (see below).

NN defines “complexity” in the way that complex-systems theory does. A system is complex if it has many parts that are interacting and integrated, hierarchically arranged, and the system’s functions cannot be reduced to the sum of the functions of the individual parts. Because NN says high complexity involves many recurrent interactions, and thus a high **ϕ** value, it defines complexity in essentially the same way as IIT does (see Note 6 in Appendix A).

**Figure 3:**
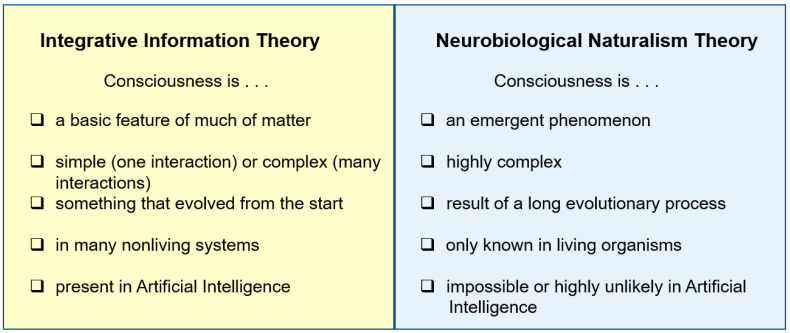
Further comparison of Integrated Information Theory and the Neurobiological Naturalism Theory, contrasting their conclusions. Furthermore, here is how the theories address the mind-body problem (or the “hard problem”): IIT nullifies the problem by starting with experience then incorporating the physical; NN is grounded in the physical, rejects all conceivability arguments, then says the problem of experience is scientifically manageable so it will be solved through the scientific method.

NN views consciousness as one of the most highly derived and complex things there is, the opposite of IIT’s view that it is fundamental enough to be present in inanimate matter and can be simple enough to characterize as one bit of information (Figure 3). As a step in addressing the complexity of consciousness, NN parses it into three main aspects. The first two are *exteroceptive consciousness* of the sensed external world and *interoceptive consciousness* of one’s sensed inner body, with these two aspects together called *image-based consciousness*. The third aspect is the *affective consciousness* of emotions and moods. Consciousness and its mechanisms must be complex, according to NN, in order to perform its many functions that contribute to an animal’s survival [70] (pp. 101–103), [82]. These functions include organizing large amounts of incoming sensory information into a unified image for choosing and directing one’s actions, while assigning strong emotions to the most important stimuli so those stimuli have the most influence on which actions to choose.

NN has additional reasons for interpreting consciousness as complex and emergent rather than simple and fundamental (fundamental means that one, universal “consciousness force” exists [83]). The first reason is that the brain is a complex organ and alterations or injuries to its complex functions affect or disrupt consciousness, so the most straightforward deduction is that consciousness is complex. This holds despite philosophical and panpsychist cogitations to the contrary. Second, our recent analysis [73] found that consciousness has all the hallmark features of a complex emergent phenomenon. That is, like all emergent phenomena it depends on *many interacting parts*; is an *aggregate system-process* that is not present in the parts alone; it arises in a (neural) system that has *hierarchical levels*, with *novel features* at each successive level; and the extrinsic environment plus its intrinsic properties impose *constraints* on what a conscious system can do.

The final way in which consciousness reveals itself to be a typical emergent phenomenon is that it arises from *multiple, alternative routes* [84] (p. 181), [85]. One example of this is how in mammals, the image-based aspect of consciousness mostly involves a different part of the brain (cerebral cortex) than does its affective aspect (subcortical regions) [70,86]. Additionally, these two aspects of consciousness seem to depend on different kinds of neural substrates: a substrate that is topographically mapped for image-based consciousness [87,88] versus a substrate that is based on four “circuit motifs for valence coding” for affective consciousness [71]. Again, these are multiple routes to consciousness.

### 3.2. Derivation of the Neurobiological Naturalism Theory

NN theory approaches the question of consciousness in a way that shows its physicalist nature and its heavy use of evidence [17,72,73]. Its derivation is shown in flow-chart form in Figure 1B. Although this derivation was primarily inductive, it needed a reasonable starting point from which to gather its specific observations. For this, it starts with several *premises* (green box 1 in the figure). These premises are tentative hypotheses (formerly called assumptions) that seem reasonable but, we realize, can be refuted or upheld by future discoveries.

*Premise 1, on image-based consciousness.* For image-based consciousness, we reasoned that any organism that demonstrably encodes topographical maps of its surrounding environment and its body—obtained from numerous senses, such as vision, touch, smell, taste, and hearing—will experience the unified mapped representation consciously. It seemed reasonable to deduce “that if a brain or body expends the energy to assemble and integrate such detailed maps, then it does use them, say, as mental reference images for moving and operating in the world” [17]. That is, the reason topographic maps evolved was for the survival advantage of being able to pinpoint the location of specific stimuli in space. Implication of Premise 1: Multisensory spatial representations are required for consciousness.

*Premise 2, on affective consciousness.* We reasoned that affective consciousness exists in any organism that is capable of complex operant conditioning [89], because such “learning from experience” accompanies positive and negative emotions in humans [5] (pp. 152–154). We adopted this premise “because it seems to give *double* evidence that an animal has emotional feelings. That is, the existence of emotion is suggested by both (1) the initial attraction to a reward, and (2) recalling the reward to motivate behavior” [17] (p. 470). Implication of Premise 2: Complex operant learning is required for consciousness.

Our definition of “complex operant learning” was initially rather vague, but it came to mean learning many new survival behaviors from experience based on rewards and punishments [72]. Now, in my opinion, it is stated even better in the Unlimited Associative Learning (UAL) concept of Ginsburg and Jablonka [41], which was recently upgraded [90] as follows: UAL says an animal is conscious if it can learn from compound, novel stimuli; learn from what it already has learned and then do so again and again; learn by trace conditioning; and perform “valence switching,” meaning the animal can learn to like (approach) what it formerly disliked (avoided) and vice versa.

A third premise could be added:

*Premise 3: All known conscious entities are alive.* I purposely stated this premise in a way that avoids the question of whether life is an absolute *requirement* for consciousness. That question is addressed in Section 3.4 on artificial intelligence and machine consciousness.

NN’s several premises do mean that it, like Integrated Information Theory, started with theoretical reasoning. But an important difference is that our premises are fewer and simpler than the many axioms and postulates of IIT; and unlike axioms, our premises are themselves testable. Obvious tests, for example, are to keep on observing how interfering with mapped sensory pathways affects image-based consciousness [91] and to assess whether UAL is always accompanied by consciousness in humans [92].

The next step in NN’s construction was to start the evidence-gathering (box 2 in Figure 1B). That is, we searched all the living organisms to find those taxa that fit the criteria of the premises. The only organisms that met all the sets of criteria are the vertebrates, arthropods, and most cephalopod mollusks (squid, cuttlefish, octopus). These three taxa must have evolved consciousness independently of each other because phylogenetic reconstruction indicates their last common ancestor was a simple worm with no brain [5] (Chapter 4), [93]. Next we scrutinized the biology of the three taxa to find other features they share that logically relate to consciousness (box 3 in Figure 1B). As shown in Table 1, we found many such convergences including: elaborated sensory organs and brains; hierarchical neuronal pathways with recurrent communications between the levels; the proposed causal mechanism of synchronized oscillations; active lifestyles; and brain mechanisms for directing attention and storing memories [73]. This set of features is not present in other animals, such as worms, clams, and sea urchins. Although we named the shared features the “special neurobiological features of consciousness,” they are actually our version of the above mentioned neural correlates of consciousness (NCCs) [7,9] in a work-in-progress form.

The neural-correlates approach to investigating consciousness has been criticized as doing a bad job of identifying *causes*, especially of how the neural mechanisms could cause feelings [9] (pp. 71–73), [18] (p. 2), [26] (p. 87). In my view, however, Searle successfully answered this criticism by framing his Biological Naturalism as a scientific problem, and his words also apply to our Neurobiological Naturalism (box 4 in Figure 1B):

The typical pattern in science has consisted of three stages. First we find correlations [the neural correlates of consciousness or NCC]... The second step is to check to see whether or not the correlation is a genuine causal correlation... The usual tests for causation, as applied to this problem, would be, first, can you produce consciousness in an unconscious subject by producing the NCC, and, second, can you shut down the consciousness of a conscious subject by shutting down the NCC? All of this is familiar scientific practice. The third step, and we are a long way from reaching this step, is to get a general theoretic account... Why should these causes produce these effects?

[77] (p. 172)

This is the recipe for discovering more and more on how neuronal mechanisms generate experience. It is similar to the “natural kinds approach” of Shea and Bayne [21,94] for scientifically finding the correlates of consciousness in order to reveal the underlying mechanisms.

NN also addresses the mind-body problem, and in a physicalist way [6,73]. Besides saying scientific progress will reveal the physical mechanisms by which nervous systems produce or constitute consciousness, NN shows that two major barriers between objective observation and subjective experience are already explainable as physical: As an outside observer I do not have access to your brain processes in your body, nor do I as a conscious subject have access to the neuronal mechanisms that cause my experiences. In both cases the physical connections are just not there.

Approaches based on neural correlates of consciousness have also been criticized [26] for not addressing the “hard problem” and related philosophical issues [83]. How does NN answer this criticism? My answer is that the “hard-problem”, as stated by Chalmers [22], comes from dubious premises that rig the exercise *a priori* to make a scientific solution impossible. One major premise is that “philosophical zombies” can exist as perfect duplicates of humans except they lack consciousness, to which the scientist responds, not in the real world unless the claimants show that such fantastic beings can exist [18]. In science, the burden of proof is on those who make extraordinary claims, this rule being necessary to prevent giving undue credibility to all forms of pseudoscience [95]. The case against there being a hard problem is summarized by Klein and Barron as:

... we are in Chalmers’ [96] taxonomy committed Type-C Materialists. We are unimpressed by conceivability arguments [that zombies could exist]. We think that scientific advances will show where such arguments go wrong, as they have in other scientific domains.

[97] (p. 2)

### 3.3. Neurobiological Naturalism is an Inductive Theory, But of What Value?

Is inductive inference as used by NN preferable to deductive inference as used by IIT? One could argue that both are equally effective for scientifically investigating consciousness not only because scientific inquiry freely uses both deduction and induction, but also because their weaknesses balance out. That is, the deductive IIT is susceptible to errors that arise from not confirming all its theoretical steps with evidence (Section 2.2.1), but the inductive NN is equally susceptible to errors that arise when its specific observations are wrong, biased, weighed incorrectly, misinterpreted, missed, or unavailable. I disagree that the two approaches are equal and say that the inductive inference used by NN is better because it is more self-correctable. This correctability is due to science’s (and NN’s) requirement for constant retesting at every important step. The evidence that arises from the retesting will expose inconsistencies when these appear and therefore will spotlight wrong interpretations. These frequent checkpoints should prevent erroneous ideas from accumulating. As argued above, this continual self-correction does not occur in theory-heavy forms of deductive inference that suffer from the Empirical Evaluation Delay.

NN is not yet a formal or quantitative theory but its inductively derived results can be tied together into a cohesive set, which I call the “NN Theory to this point.” This set offers physicalist explanations for:

- how consciousness is a classic example of an emergent property of complex systems (Section 3.1);
- which organisms have consciousness (vertebrates, arthropods, cephalopods), and, by extension, the time when consciousness first evolved (about 550 million years ago, when the fossil record shows the vertebrates and arthropods had diverged [70]);
- a new list of NCCs, as shared by these animal taxa (Table 1). Many of these NNCs differ from those offered previously, which mostly include mammalian, neocortical features [98];
- a route to determine which of these NCCs generate consciousness, and how this happens, by applying Searle’s recipe that uses the scientific method (Section 3.2);
- how a reconsideration of the known physical barriers can solve some aspects of the mind/body problem (Section 3.2 and [73]).

This formulation of NN provides a map for answering the central questions about consciousness in the future. In the meanwhile, here are NN’s current predictions along with ways to test them. Like other theories [11,99,100,101], NN predicts that synchronous electrical oscillations in the brain indicate consciousness (Table 1). Therefore, it can be tested by recording more electrical oscillations and recurrent neural signaling in the brains of fish, crabs, and octopuses, both before and after perturbational interventions (e.g., masking [102]); and NN can be falsified if these features are not found in these animals. NN can also be tested by investigating certain invertebrates that seem to be at the cusp of consciousness according to NN’s predictions: some land snails and polychaete worms called alciopids, both of which have relatively well-developed eyes [103,104]. Here, the prediction is that all the neural correlates (Table 1) will vary together as a set, meaning, for example, that animals with the most elaborate sensory organs will also have the most complex brains, the most memory capacity, and the most valence-coding centers in their brains. Any dissociation (e.g., large brain but little memory or valence coding) could falsify the NN theory.

Ultimately, the relative merits of IIT and NN will be determined by which theory has the most explanatory power, for all the available evidence on consciousness. For now, however, the less-mature NN does not make many of the kinds of predictions that can be evaluated against those of IIT. The reason such comparative evaluation is difficult is that IIT’s predictions focus on the human cerebral cortex but NN’s predictions focus on nonmammalian animals that lack a cerebral cortex. Even though the two theories are difficult to compare empirically, there is one point on which they differ strongly, and which ultimately could be used to judge which theory is more valid. That difference is IIT’s interpretation of consciousness as fundamental and simple (in a photodiode), versus NN’s interpretation that it is emergent and highly complex (only in elaborate brains).

### 3.4. Artificial Consciousness

NN and IIT differ in their answers to the often-asked question of whether artificial intelligence can be conscious (Figure 3). IIT answers yes, because it says anything built with interacting parts has some consciousness [31] (Section 4a), [105]. The only limitation IIT imposes is that the designed network cannot be the strictly feedforward type, as in most existing digital computers [9]. Recurrent computer networks do exist [106,107], however, and they fit IIT’s criterion for artificial consciousness.

By contrast, NN is resistant to the idea of artificial consciousness. Because consciousness, as traditionally understood, only exists in living organisms, both Feinberg and I basically believe that consciousness needs life. However, we have a point of disagreement. Feinberg flat-out insists that life is necessary because that is literally how NN was defined (personal communication), whereas I am willing to allow that someday consciousness could be achieved by an ultra-advanced machine. However, that future machine would have to match natural consciousness in all its complexity and efficiency. Not only must it incorporate all the special features of Table 1, it would also have to integrate all the classes of sensory stimuli it receives (visual, auditory, tactile, chemosensory) in a way that allows the machine to move, find its own sustenance, survive in nature, reproduce itself, and adapt to new environments over its generations—in accordance with the functions of consciousness that have been identified [70] (pp. 101–103). No machine could achieve these things in the foreseeable future of human technological progress (millennia?). That means artificial consciousness is not on the horizon so I agree with Feinberg that consciousness needs life, for all practical purposes. In this view, those who predict that human-like artificial consciousness is right around the corner [108,109] underestimate the complexity of the phenomenon.

On the other hand, this is not meant to cast down the ongoing attempts to develop artificial consciousness. It is possible that consciousness is not as complex or impenetrable as NN implies, so we may discover its principles by looking outside the box of living systems, at machine learning. Consciousness may be like flying, in which an airplane design achieves flight more simply than a living bird does. One who is optimistic about artificial consciousness could also point out that scientific advances have successfully explained the mystery of “life” to the point where vitalism is no longer credible, and “life” once seemed just as puzzling as consciousness does today.

### 3.5. Potential Weaknesses of Neurobiological Naturalism

The NN theory has some limitations. Three of these were already presented: first, that NN is less developed and less theoretically formal than IIT; second, like all theories based on inductive inference, NN’s observational “data” could be incomplete or incorrectly interpreted; and third, unlike IIT, NN does not have a built-in solution to the mind-body problem, though it does offer a path to a scientific solution (see Section 3.2). Fourth, NN is more difficult to test technically than are most other theories because it does not focus on humans. Here is why: the gold standard of consciousness studies is to present subjects with stimuli they can interpret either as conscious or unconscious depending on the experimental conditions; e.g., with paradigms called the attentional blink, binocular rivalry, flash supression, and other forms of masking [8,110]. This works for human subjects, monkeys, and even intelligent crows [111], but the fish and insects of interest to NN are not expected to be cooperative or trainable enough to participate in such demanding and confining laboratory tests.

A fifth potential problem with NN involves its unique NCCs. The many other studies that strive to pin down the NCCs already disagree about these correlates within the relatively narrow taxonomic clade of humans and other mammals with a cerebral cortex [9] (pp. 58–67), but our NN brings in quite different correlates that fit the alien brains of squids and crabs (Table 1). The investigators who seek the NCCs strive for a consensus so they can start testing the NCCs to find the mechanisms behind consciousness. NN’s discordant NCCs drive the endeavor farther away from this consensus.

Sixth, NN’s conclusion that the arthropods and “lower” vertebrates are conscious will seem wrong to some investigators, although many who study animal consciousness have come to this same conclusion [112,113,114,115,116,117]. The most prominent alternate view is that consciousness is confined to mammals with their cerebral cortex (and probably to birds, who have analogous parts of their neopallium) [5,12,111]. The need for a cerebral cortex certainly remains the dominant claim in the heavily *human*-centered literature on consciousness, and some investigators still say that only humans have consciousness [118,119], but refuted in [120].

Therefore, NN runs into the problem of widespread disagreement about when consciousness emerged in phylogeny and also into the philosophical problem of “discontinuity,” or how consciousness could have appeared abruptly at some point in the evolution of a lineage [35] (Section 3.3), [121]. NN can successfully answer the discontinuity challenge because it incorporates Emergence Theory, which itself was designed to explain how novel features can arise (see above). However, IIT does this even better than NN does, because IIT avoids the discontinuity problem entirely by starting consciousness at the simplest elementary particles.

## 4. Conclusions

This paper examined Integrated Information Theory and compared it to Neurobiolical Naturalism Theory, two dissimilar approaches to explaining consciousness. I compared many different aspects of the two theories but primarily compared the ways they are constructed (Figure 1). IIT, with its creative, phenomenology-first slant, its compelling axioms, and its laudible mathematical formalism, is fundamentally a theory-driven approach based on deductive inference. NN, on the other hand, with its comparative evolutionary aspects that are devoted to assembling NN’s own neural correlates of consciousness, is fundamentally an observation-driven approach and is based on inductive inference.

Both deductive and inductive approaches are valuable in scientific investigation, but is one better than the other in the particular case of investigating consciousness? Proponents of the deductive approach can criticize the inductive approach as not having enough theory to guide it, and proponents of the inductive approach can criticize the deductive approach as not using enough supporting evidence to avoid and correct its errors. Scientific methodology has a recipe for building theories that avoids both of these criticisms. That recipe is to use short, alternating steps of theorizing then evaluating with hard evidence, usually stated as hypothesize-test-hypthesize-test, etc. IIT does not follow this recipe and its long stretches of unchecked theorizing could be a weakness of the theory that I named an Empirical Evaluation Delay. Therefore, recognizing that IIT is a deductive theory and NN is an inductive one does not show them to be equally valid approaches in the eye of traditional scientific methodology.

Recognizing that NN is an inductive theory, however, has helpful implications. It shows that NN is still in its formative stages, less advanced than I as a founder of NN had thought it was. This points to valuable new directions for NN to take going forward: NN can summarize its findings more formally (begun with List A–E in Section 3.3), it can examine its founding observations more closely, and it can do more experimental testing. This fits the main conclusion of this paper, that the scientific theories of consciousness can be more evidence-driven in the future.

## Figures and Tables

**Table 1:** The neural correlates of consciousness according to Neurobiological Naturalism Theory: the special features that are shared by vertebrates, arthropods, and cephalopods. Mostly from [73].

Neural complexity (more than in a simple, core brain) Brain with many neurons (>100,000?) Many subtypes of neurons Elaborated sensory organs Eyes, receptors for touch, taste, hearing, smell Neural hierarchies with neuron–neuron interactions Extensive reciprocal communication in and between pathways for the different senses Brain’s neural computing modules and networks are distributed but integrated, leading to local functional isolation plus global coherence Synchronized communication by brain-wave oscillations Neural spike trains form representational codes The higher brain levels allow the complex processing and unity of consciousness Higher brain levels exert considerable influence on the lower levels, such as motor neurons, for top-down causality Hierarchies that let consciousness predict events a fraction of a second in advance Pathways that create mapped mental images or affective states Neurons are arranged in topographic maps of the outside world and body structures Valence coding of good and bad, for affective states Feed into pre-motor brain regions to motivate, choose, and guide movements in space for high mobility Brain mechanisms for selective attention and arousal Memory of perceived objects or events

## Data Availability

Not Applicable.

## Appendix A

1. In IIT, this relationship between the physical and the experience is not *directly* causal. Rather, if the physical elements specify a maximally irreducible, specific, compositional, intrinsic cause-effect power, also called a “MICS” or just a “causal structure,” then that cause-effect power is *identical* to an experience and everything within that experience must be accounted for in causal terms. Physical elements are the substrate of consciousness, but do not cause it. Instead, if they have the appropriate causal properties, then that causal power will be identical to consciousness, but the substrate itself will not be (Robert Chis-Ciure, personal communication).
2. Hard problem and explanatory gap mean basically the same thing, the hard problem just being the difficult problem of bridging the gap. Both ask how physical processes could ever be accompanied by any experience, and by different experiences, such as “red” versus “green”.
3. Again, the MICS is a calculated, abstract representation of an experience and the experience’s subparts in multidimensional space [2]. It *is* the experience, according to IIT.
4. A note is in order on the *types* of evidence to which I refer. Along with the evidence of *objectively* observable facts, science could probably use and investigate the “phenomenal facts” of experiences, as long as such “facts” seem consistent across different individuals. An example of a phenomenal fact is that the loudness level one experiences roughly doubles with each 10-decibel increase in sound intensity [122]. However, I did not find that the construction of IIT used any phenomenal facts beyond its axiom/postulate stage—it just used math and logic. Thus, I am only considering how IIT uses objective, physical evidence.
5. The computational difficulty of ramping up from a simple system with just a few nodes to the “intermediate” level of the *C. elegans* nervous system is that IIT’s Transition Probability Matrix would be very hard to calculate. *C. elegans* has 302 neurons (nodes), so all these elements must be perturbed in all possible states, and all possible state transitions must be recorded. Even for just ON/OFF (firing/silence) states of neurons, for 302 nodes there are 8.148 × 10^90^ possible states. One must compute the probability for each of these states to transition into any other one to kickstart the IIT analysis, and that is far beyond the capacity of existing computers. However, there may be an empirical way around this problem. Marshall et al. [63] argued that the desired, **ϕ** parameter scales almost linearly with the amount of differentiation of a system’s potential states, which is measured as ***D***. This ***D*** is easier and faster to compute than is **ϕ** and might be used as a proxy for **ϕ** in the intermediate systems (Robert Chis-Ciure, personal communication).
6. Absolute measures of complexity are difficult to obtain, so NN often uses relative measures instead. For example, according NN’s analysis in Section 3.2 the roundworm *C. elegans* is not conscious with its brain of 140 neurons, but arthropods are conscious with their brains of ≤100,000 neurons [5] (Chapter 9). Thus NN uses a relative measure to say the lower limit for consciousness is a brain that is more complex than the worm’s but less complex than the arthropod’s.
